# *Bothrops jararaca* Peptide with Anti-Hypertensive Action Normalizes Endothelium Dysfunction Involved in Physiopathology of Preeclampsia

**DOI:** 10.1371/journal.pone.0023680

**Published:** 2011-08-17

**Authors:** Gabriel Benedetti, Katia L. P. Morais, Juliano R. Guerreiro, Eduardo Fontana de Oliveira, Mara Sandra Hoshida, Leandro Oliveira, Nelson Sass, Ivo Lebrun, Henning Ulrich, Claudiana Lameu, Antonio Carlos Martins de Camargo

**Affiliations:** 1 Center for Applied Toxinology-Centros de Pesquisa, Inovacio e Difusao, Instituto Butantan, Sao Paulo, Brazil; 2 Departamento de Bioquímica, Universidade Federal de São Paulo, Sao Paulo, Brazil; 3 Laboratório de Bioquímica de Proteínas, Centro de Biotecnologia Agrícola, Departamento de Ciências Biológicas, Escola Superior de Agricultura “Luiz de Queiroz”, University of Sao Paulo, Sao Paulo, Brazil; 4 Laboratório de Fisiologia Obstétrica, Faculdade de Medicina, Universidade de São Paulo, Sao Paulo, Brazil; 5 Hospital Maternidade Vila Nova Cachoeirinha, Sao Paulo, Brazil; 6 Laboratório de Bioquímica e Biofísica, Instituto Butantan, Sao Paulo, Brazil; 7 Departamento de Bioquímica, Instituto de Química, Universidade de São Paulo, Sao Paulo, Brazil; 8 Department of Cell and Developmental Biology, Institute of Biomedical Sciences, University of São Paulo, Sao Paulo, Brazil; University of Bristol, United Kingdom

## Abstract

Preeclampsia, a pregnancy-specific syndrome characterized by hypertension, proteinuria and edema, is a major cause of fetal and maternal morbidity and mortality especially in developing countries. *Bj*-PRO-10c, a proline-rich peptide isolated from *Bothrops jararaca* venom, has been attributed with potent anti-hypertensive effects. Recently, we have shown that *Bj*-PRO-10c-induced anti-hypertensive actions involved NO production in spontaneous hypertensive rats. Using *in vitro* studies we now show that *Bj*-PRO-10c was able to increase NO production in human umbilical vein endothelial cells from hypertensive pregnant women (HUVEC-PE) to levels observed in HUVEC of normotensive women. Moreover, in the presence of the peptide, eNOS expression as well as argininosuccinate synthase activity, the key rate-limiting enzyme of the citrulline-NO cycle, were enhanced. In addition, excessive superoxide production due to NO deficiency, one of the major deleterious effects of the disease, was inhibited by *Bj*-PRO-10c. *Bj*-PRO-10c induced intracellular calcium fluxes in both, HUVEC-PE and HUVEC, which, however, led to activation of eNOS expression only in HUVEC-PE. Since *Bj*-PRO-10c promoted biological effects in HUVEC from patients suffering from the disorder and not in normotensive pregnant women, we hypothesize that *Bj*-PRO-10c induces its anti-hypertensive effect in mothers with preeclampsia. Such properties may initiate the development of novel therapeutics for treating preeclampsia.

## Introduction

Preeclampsia, a pregnancy-specific syndrome characterized by hypertension, proteinuria and edema, causes fetal and maternal morbidity and mortality with high incidence in developing countries [1]. Symptoms of preeclampsia are currently combated by sodium restriction, rest and medication for blood pressure control to avoid complications for the mother and prolong the pregnancy for fetal maturation [2]–[4]. However, this attempt is rather unspecific with possible side effects for the developing fetus [5]–[7]. Currently, the only therapy of preeclamsia involves placenta removal resulting in pre-term birth [8]. Therefore, novel drug development for pregnancy-specific conditions remains a challenge [7]. Our current knowledge is that hypertension in preeclampsia is secondary to placental underperfusion. Thus, therapeutic reduction of systemic blood pressure is not believed to reverse the primary pathogenic process, and antihypertensive medication has never been demonstrated to cure or reverse preeclampsia [3].

The pathology of preeclampsia has been characterized by systemic inflammation, oxidative stress, alterations in the levels of angiogenic factors, and vascular reactivity leading to hypertension of the mother and metabolic alterations in the fetus [8], [9]. A number of evidence suggests that the clinical manifestations are caused by endothelial malfunction including insufficient production of nitric oxide (NO) [10], [11]. NO production originates from the action of endothelial nitric oxide synthase (eNOS), a Ca^2+^-dependent enzyme, using L-arginine as substrate [12]. This gaseous messenger then diffuses from endothelial cells to vascular smooth muscle thus providing a dilator tone [13]. It has been suggested that the concentration of L-arginine in the pregnant plasma, activity and levels of eNOS which is constitutively expressed, but not the inducible NOS are altered in endothelial cells and play critical roles in pathogenesis of preeclampsia including vascular stress and inflammatory processes [9], [14], [15], since the low availability of L-arginine uncouples eNOS activity, decreases NO production and increases eNOS-dependent superoxide generation [9], [15]. Therefore, it is expected that the sustained concentration of L-arginine in endothelial cells is likely to play a critical role not only in the control of systemic blood pressure but also in inhibition of inflammatory processes [16], [17].

A family of pyroglutamyl proline-rich oligopeptides has been isolated from the venom gland of the pit viper *Bothrops jararaca* (*Bj*-PROs) and many of those showed strong bradykinin-potentiating activity. Recent results indicated the targets of *Bj*-PRO-10c, resulting in neurotransmitter release, NO production and alteration of baroreflex control, resulting in alteration of arterial pressure and heart rate [18], [19].

Our group has recently demonstrated that *Bj*-PRO-10c augments the L-arginine levels *ex vivo* and *in vivo*, due to activation of argininosuccinate synthase (ASS), a rate limiting enzyme in L-arginine biosynthesis in the NO-citrulline cycle for NO production in the endothelium [20].

In view of the absence of molecular targets for combating preeclampsia which would allow to control arterial pressure levels in hypertensive pregnant without the risk of inducing sharp drops in blood pressure and heart rate of the fetus, we have investigated in the present study, whether the anti-hypertensive activity of peptide *Bj*-PRO-10c would correct dysfunction of human umbilical vein endothelial cells from pregnant women suffering from preeclampsia (HUVEC-PE). In fact, in the presence of *Bj*-PRO-10c NO production by HUVEC-PE was similar to HUVEC from normotensive pregnants. Together with augmented NO production, bioavailability of L-arginine for eNOS re-coupling increased, leading to decreased superoxide generation and turning *Bj*-PRO-10c into a promising tool for preeclampsia resulting from disturbed NO metabolism.

## Results

### *Bj*-PRO-10c-induced NO production by HUVEC-PE

**Figure 1:**
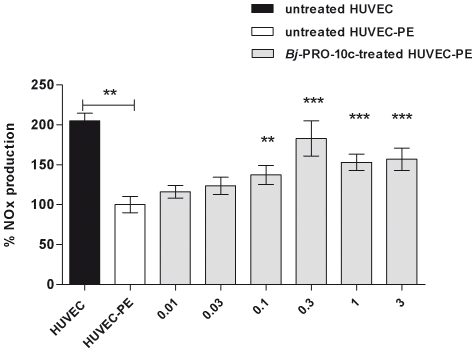
*Bj*-PRO-10c-induced NO production in HUVEC-PE. HUVEC-PE cultures were incubated in serum-free medium for 24 h with increased concentrations of *Bj*-PRO-10c. Control experiments were made with HUVEC in the absence of *Bj*-PRO-10c. NO products (NOx) of untreated HUVEC, HUVEC-PE and *Bj*-PRO-10c-treated HUVEC-PE were measured in by a chemiluminescence assay. Basal NO production by HUVEC-PE was considered as 100%. The shown data are mean values normalized for protein content ± S.E. of six experiments. ** *P*<0.01 and *** *P*<0.001 compared to untreated HUVEC-PE.

Basal NOx production by HUVEC was twice higher than compared to HUVEC-PE (Figure 1), supporting the hypothesis that NO deficiency is involved in the pathophysiology of preeclampsia [10], [11]. In another study [19], we have shown that *Bj*-PRO-10c-induced anti-hypertensive actions involved NO production in spontaneous hypertensive rats. Therefore, we studied the effects of various *Bj*-PRO-10c concentrations (0.01 to 3 µM) on NO production of HUVEC-PE following exposure to the peptide for 24 h at 37°C. *Bj*-PRO-10c dose-dependently augmented NO production in HUVEC-PE reaching maximal responses (83% of NO levels of normal HUVEC) at 0.3 µM peptide concentration (Figure 1).

### Increased L-arginine production by endothelial cells in the presence of *Bj*-PRO-10c

**Figure 2:**
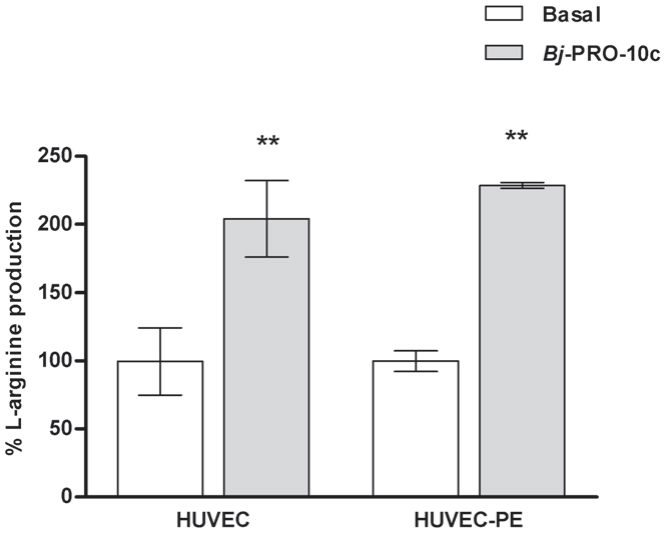
*Bj*-PRO-10- induced L-arginine production in endothelial cells. HUVEC and HUVEC-PE were incubated in serum-free medium in the absent or presence of *Bj*-PRO-10c. The medium was collected, and the cells were lysed for determination of L-arginine levels by HPLC analysis. L-Arginine production levels in the absence of *Bj*-PRO-10c were considered as 100%. The data are presented as mean values normalized for number of cells ± S.E. of three independent experiments. ** *P*<0.01 compared to basal production.

*Bj*-PRO-10c was recently described as an activator of ASS activity, the step-limiting enzyme in the L-arginine biosynthesis in the NO-citrulline cycle for NO production in the endothelium [20]. In the same work, authors also showed that the peptide caused an increase in L-arginine production in a kidney cell line (HEK293 cells) [20]. L-arginine in endothelial cells is likely to play a critical role in the control of the systemic blood pressure and the inflammatory process, both affected in preeclampsia [16], [17]. We have observed that the basal L-arginine production levels in HUVEC-PE were 25% lower than in HUVEC (data not shown). In addition, 24 h incubation of the endothelial cells with 0.3 µM *Bj*-PRO-10c increased L-arginine production by HUVEC and HUVEC-PE by 104% and 118%, respectively (Figure 2).

### Decreased superoxide production in HUVECs-PE in the presence of *Bj*-PRO-10c

**Figure 3:**
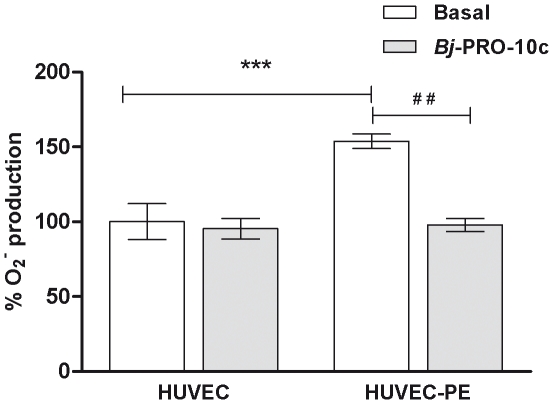
Inhibition of superoxide production in HUVEC-PE by *Bj*-PRO-10c. HUVEC and HUVEC-PE were pre-incubated in serum-free medium for 24 h in the absence or presence of 1 µM *Bj*-PRO-10c. After this period of pre-incubation the cells were treated with the superoxide-sensitive fluorescent probe and emitted fluorescence was measured by using an automated plate reader. Superoxide production by HUVEC was considered as 100%. The data are expressed as the mean values normalized for number of cells ± S.D. of three independent experiments. *** *P*<0.001 compared to HUVEC basal production and ^##^ *P*<0.01 compared to HUVEC-PE basal production.

Limited bioavailability of NO, either because of a decreased formation of NO due to low viability of substrate for NOS, or increased scavenging of NO by superoxide, can result in reduced vasodilatation or induce inflammatory processes observed in preeclampsia [15], [21]. We have previously shown that *Bj*-PRO-10c is able to promote substantial increase of L-arginine and NO production in HUVEC-PE. We then hypothesized whether a likely improvement of the availability of NO induced by *Bj*-PRO-10c would reduce the oxidative stress of the endothelial cells of preeclamptic women. The results presented in Figure 3 demonstrate that the production of superoxide by HUVEC-PE was 50% higher than in HUVEC. In *Bj*-PRO-10c-treated HUVEC-PE superoxide production was substantially reduced by approximately 50%, while *Bj*-PRO-10c did not elicit any changes in superoxide levels in HUVEC from normotensive women (Figure 3). The results obtained herein confirm that this peptide exerts protective functions against preeclampsia-related accumulation of reactive oxygen species.

### Characterization of *Bj*-PRO-10c-induced [Ca^2+^]*_i_* transients in HUVEC and HUVEC-PE

**Figure 4:**
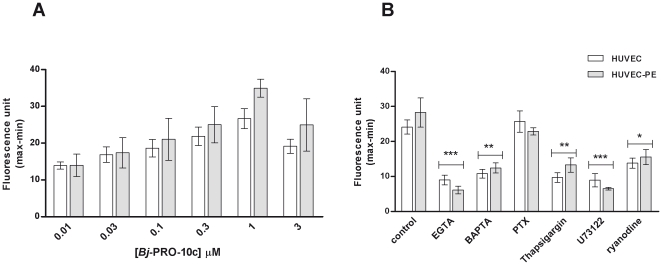
Characterization of *Bj*-PRO-10c-induced [Ca^2+^]*_i_* elevations in endothelial cells. **A.** Changes of maximal peak heights of [Ca^2+^]_i_ responses induced by increasing concentrations of *Bj*-PRO-10c (0.01–3 µM) were measured in HUVEC and HUVEC-PE by microfluorimetry. **B.** Evaluation of *Bj*-PRO-10c-induced [Ca^2+^]_i_ transients in HUVEC and HUVEC-PE in the presence of chelating agents and specific inhibitors of Ca^2+^ signaling. Cells were pre-incubated for 5 or 30 min with 10 mM EGTA or 10 µM BAPTA (extracellular and intracellular Ca^2+^ chelators), respectively. Cells also were pre-treated for 18 h with 100 ng/ml pertussis toxin (PTX) for inhibition of G_i/o_-protein mediated receptor responses and for 30 min with U73122, a PLC-γ inhibitor. Calcium release from intracellular stores was inhibited by a 30 min pre-incubation of cells with 200 ng/ml thapsigargin. The participation of ryanodine-sensitive calcium stores was studied following 30 min pretreatment of cells with 50 µM ryanodine. * *P*<0.05, ** *P*<0.01 and *** *P*<0.001 compared to *Bj*-PRO-10c control data obtained in the absence of pre-treatment. The shown data are mean values ± S.E. of five independent experiments.

Endothelial NOS (eNOS) is a Ca^2+^-dependent enzyme that catalyzes NO formation in the endothelium, whose activity is decreased in preeclampsia [14], [15]. Therefore, increases in [Ca^2+^]*_i_* are important for NO production by endothelial cells. *Bj*-PRO-10c-induced elevations of [Ca^2+^]*_i_* in both HUVEC and HUVEC-PE, reaching peak values at 1 µM peptide concentration (Figure 4A). Mechanism of induction of [Ca^2+^]*_i_* fluxes in HUVEC and HUVEC-PE by *Bj*-PRO-10c were investigated using specific inhibitors of G-protein coupled receptors and their signal transduction (Figure 4B). Pretreatment with 100 ng/ml of pertussis toxin, an inhibitor of G_i/o_-protein-coupled receptor activation, did not affect *Bj*-PRO-10c-induced [Ca^2+^]*_i_* mobilization, neither in HUVECs nor in HUVECs-PE, while preincubation with U-73122, a specific inhibitor of PLC-γ activity, inhibited the *Bj*-PRO-10c-promoted [Ca^2+^]*_i_* increase. Moreover, pretreatment of HUVEC and HUVEC-PE with 50 µM ryanodine, used for inhibition of calcium-induced calcium release (CICR) mechanisms, resulted in a significant reduction of *Bj*-PRO-10c-evoked [Ca^2+^]*_i_* transients. *Bj*-PRO-10c-provoked [Ca^2+^]*_i_* responses were affected in the presence of EGTA and BAPTA-AM, chelating extra- and intracellular calcium, respectively. *Bj*-PRO-10-c-induced responses were also blocked following preincubation of cells with thapsigargin, inhibiting endoplasmic reticulum Ca^2+^-ATPase [22] and leading to depletion of intracellular calcium stores, confirming the participation of both, calcium influx into the cell and intracellular calcium stores.

### Modulation of ASS and eNOS expression by *Bj*-PRO-10c

**Figure 5:**
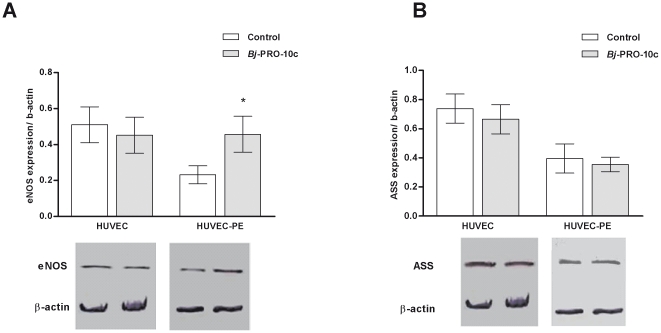
Analysis of eNOS and ASS protein levels in HUVEC and HUVEC-PE. Endothelial cells were incubated for 24 h in serum-free medium in the absence (control) or presence of 1 µM *Bj*-PRO-10c. Then, cells were lysed and 50 µg of cell homogenate proteins were used for Western-blot analysis as detailed in Materials and Methods. Relative quantification of eNOS (A) and ASS (B) expression levels was obtained by densitometry scanning. β-Actin immunostaining was used as endogenous control for normalizing of protein expression. Data (mean values ± S.E.) are representative for three independent experiments. * *P*<0.05 compared to untreated control HUVEC-PE.

Western-blot analysis confirmed that eNOS and ASS expression in HUVEC-PE was lower when compared to HUVEC (Figure 5A and B). When HUVEC and HUVEC-PE were pretreated for 24 h with *Bj*-PRO-10c, eNOS expression in HUVEC-PE was two-fold increased, when compared to expression levels of non-treated control HUVEC-PE. In the presence of the peptide, eNOS levels in HUVEC-PE were similar to those of HUVEC from normotensive pregnant women. However, ASS expression in HUVEC-PE and both eNOS and ASS expression in *Bj*-PRO-10c-treated HUVEC were not significantly different from non-treated cells (Figure 5A and B).

## Discussion

Preeclampsia is a syndrome in women occurring during pregnancy and is characterized by elevated blood pressure and proteinuria. The development of preeclampsia is accompanied by increased vascular reactivity as response to vasoconstrictors, decreased NO production, and increased accumulation of superoxide [23]. The results presented here provide important information regarding the use of *Bj*-PRO-10c as a possible model for the development of a therapeutic modality to treat the condition preeclampsia-eclampsia. *Bj*-PRO-10c is an oligopeptide endowed with antihypertensive activity that decreases blood pressure in hypertensive but not in normotensive rats [24]. Recently, our group demonstrated that this peptide positively modulated ASS activity *in vitro* and *in vivo*, thereby increasing NO production [20]. This fact prompted us to propose the use of *Bj*-PRO-10c for treatment of preeclampsia, since this peptide should not affect blood pressure of the fetus, a problem with drugs currently used for minimizing health problems arising from preeclampsia. However, the peptide could correct the deficiency in NO production observed in specific tissues of preeclamptic pregnancy, as observed in the present work in human umbilical vein endothelial cells (HUVEC).

Guerreiro and co-workers showed that *Bj*-PRO-10c led to increases the production of NO in HUVEC due to activation of the ASS, the rate-limiting enzyme for the continuous regeneration of L-arginine from L-citrulline in the NO-citrulline cycle, thus providing NOS with sustained supply of substrate [20]. In the present study, we found that NO production of HUVEC-PE is lower than that of HUVEC, being in agreement with many studies suggesting deficiency in NO production by endothelial cells in preeclampsia [9], [15]. *Bj*-PRO-10c was able to increase NO production in HUVEC-PE to normal physiological levels.

*Bj*-PRO-10c as is a positive modulator of ASS activity [20], was able to enhance of ASS activity of total protein of HUVEC lysed (data not shown), reflecting increased L-arginine and NO production in both, HUVEC and HUVEC-PE.

Production of reactive oxygen species, primarily superoxide anions, goes along with a deficit in NO biosynthesis considered as a determining factor in the pathophysiology of preeclampsia [15], [21]. The superoxide anion is produced by uncoupling of NOS due to the lack of its natural substrate L-arginine. In preeclampsia, excessive production of superoxide is explained by increased activity and expression of the enzyme arginase, which competes with eNOS for its substrate L-arginine. It was recently demonstrated by Sankaralingam and colleagues [25] that arginase activity was increased in the vasculature of women with preeclampsia, resulting in enhanced superoxide production. Kim and colleagues [26] demonstrated that inhibition of arginase promoted the recoupling of eNOS, failing to produce the superoxide anion and decreasing oxidative stress. Although *Bj*-PRO-10c did not affect arginase activity (data not shown), this peptide recoupled eNOS and consequently reduced the production of superoxide by HUVEC-PE, thanks to its ability of increasing ASS activity [20] and inducing L-arginine production. The eNOS is probably inactive at basal Ca^2+^ levels of resting cells, and is only activated when the intracellular calcium concentration ([Ca^2+^]*_i_*) is elevated [27], [28]. The activation of eNOS occurring through the formation of Ca^2+^/calmodulin complexes is responsible for triggering the transfer of electrons from the C-terminal to the N-terminal portion of eNOS [29], [30].

Recently it was demonstrated that *Bj*-PRO-10c is capable of inducing [Ca^2+^]*_i_* mobilization in neurons, mediated by a receptor coupled to G_i/0_-protein, followed by a calcium-induced calcium release (CICR) mechanism [18]. In the present paper we have shown that *Bj*-PRO-10c also induced [Ca^2+^]*_i_* fluxes in endothelial cells with similar mechanisms to those described in neuronal cells [18]. The depletion of intracellular Ca^2+^ stores by preincubation with thapsigargin, an inhibitor of Ca^2+^-ATPase of the endoplasmic reticulum [22], resulted in an almost complete loss of *Bj*-PRO-10c-induced [Ca^2+^]*_i_* elevations. In addition, *Bj*-PRO-10c-induced [Ca^2+^]*_i_* transients were significantly reduced in the absence of extracellular calcium. The preincubation of HUVECs with ryanodine, an inhibitor of CICR mechanism [31], significantly decreased the *Bj*-PRO-10c-induced [Ca^2+^]*_i_* response. U-73122, a specific inhibitor of phospholipase C-β (PLC-β) activtiy [32], was used to determine the involvement of this enzyme in the signaling pathway of *Bj*-PRO-10c. *Bj*-PRO-10c-induced [Ca^2+^]*_i_* increase was affected by incubation with the PLC-β inhibitor suggesting the involvement of IP_3_ formation catalyzed by the phospholipase. The preincubation of HUVECs with pertussis toxin, an inhibitor of protein G_i_ or G_o_ [33], did not cause significant reduction in *Bj*-PRO-10c-induced [Ca^2+^]*_i_* transient, suggesting that the putative receptor of *Bj*-PRO-10c did not depend on G_i_ nor G_o_ protein activation.

The data presented herein show that *Bj*-PRO-10c-induced [Ca^2+^]*_i_* transients were mediated by influx of calcium, followed by calcium release from intracellular stores sensitive to ryanodine, known as CICR mechanism. Moreover, *Bj*-PRO-10c did not promote an increase in [Ca^2+^]*_i_* in smooth muscle cells (data not shown), featuring a specificity of *Bj*-PRO-10c on endothelial cells. Such increase of [Ca^2+^]_i_ in smooth muscle cells would result in vasoconstriction and be contradictory to anti-hypertensive actions of *Bj*-PRO-10c [34].

It is known that the development of preeclampsia involves changes in expression levels of enzymes participating in NO metabolism [9], [25]. Levels of eNOS, the enzyme responsible for NO synthesis in the endothelium from L-arginine, are decreased in HUVEC-PE [9] together with the ASS expression, an observation reported here for the first time. Low eNOS and ASS protein levels could explain why NO production is impaired in HUVEC-PE. Therefore, a correction of eNOS and ASS expression rates to normal levels could contribute to the treatment of preeclampsia. Indeed, eNOS expression of HUVEC-PE increased significantly in the presence of *Bj*-PRO-10c. Although the peptide did not increase the ASS expression, it augmented the activity of this enzyme [20], thereby possibly compensating reduced protein levels. Furthermore, treatment of HUVEC with *Bj*-PRO-10c did not alter eNOS expression. Thus, although *Bj*-PRO-10c-induced signaling pathways in HUVEC and HUVEC-PE are the same, due to slight differences in signal intensity in HUVEC-PE, *Bj*-PRO-10c-promoted calcium fluxes may induce distinct pathways [35] leading to an increase in eNOS expression in HUVEC-PE, which are not activated in HUVEC from normotensive women.

Together, our work has identified eNOS and ASS as targets of *Bj*-PRO-10c for NOS production in HUVEC from pregnant women. In the presence of the peptide, eNOS expression and activity as well as ASS activity are enhanced increasing NO levels to those of normotensive women. Since *Bj*-PRO-10c promoted NO production in HUVEC from patients suffering from the disorder and not in normotensive pregnant women, it is suggested that *Bj*-PRO-10c would induce its anti-hypertensive effect in mothers with preeclampsia, such properties may initiate the development of novel specific therapeutics for the treatment of preeclampsia.

## Materials and Methods

### Ethics Statement

The study received prior approval from the Hospital Municipal Maternidade-Escola Dr. Mário de Moraes A. Silva, São Paulo, Brazil (01/2009) and Universidade Federal de São Paulo, São Paulo, Brazil (1956/2008) and written informed consent was obtained from women donors of umbilical cord.

### Synthesis and purification of *Bj*-PRO-10c

*Bj*-PRO-10c (<ENWPHPQIPP), was synthesized using an automated PSSM-8 peptide synthesizer (Shimadzu Corp., Chiyoda-ku Kyoto, Japan) by a stepwise solid-phase method using *N*-9-fluorenylmethoxycarbonyl (Fmoc) chemistry [36]. The *Bj*-PRO-10c was purified on a reversed-phase column (ODS-C18, Shimadzu, 20 mm×250 mm column) coupled to a HPLC system equipped with the binary pump LC-6AD and the SPD-10AV UV-Vis detector. Molecular mass and purity of the synthesized peptide were confirmed by MALDI-TOF mass spectrometry (Amersham Biosciences, Uppsala, Sweden) [18].

### Collection and establishment of human umbilical vein endothelial cells (HUVECs)

Umbilical cords were collected from normotensive pregnant (n = 8) and pregnants suffering from preeclampsia (n = 9). Preeclampsia was defined as new-onset hypertension (>140×90 mmHg) associated with proteinuria >0.3 g/24 h. In addition, all patients in this study had early-onset preeclampsia (<34 gestational weeks) and proteinuria was higher than 1 g/24 h in all cases. All women in the control group had normal pregnancy and delivered at term. The umbilical cords were placed on aluminum foils, checked for any injuries and then cleaned externally with 70% ethanol and sterile gauze. We conducted a cross court at its end, where it was located in the umbilical vein for cannulation. Then, with a key three-way connected to a 20 ml syringe containing sterile saline solution, the vein was washed at least three times to remove traces of blood contained within it. Immediately following the washing steps, the umbilical cord was treated for 15 min at 37°C with collagenase type IV (Worthington Biochemical Corp., NJ, USA) using 1 mg/ml enzyme for each cm of umbilical cord. The solution containing collagenase was transferred into a sterile Falcon tube followed by addition of fetal calf serum to obtain a final concentration of 10% in order to neutralize collagenase. Once neutralized, the solution was centrifuged for 10 min at 3,000 g at 4°C; the supernatant was discarded and cells were resuspended in 2 ml of complete culture medium (Human Endothelial culture medium supplemented with 10% FBS, 1% L-glutamine, 1% penicillin/streptomycin, 1% gentamicin and 1% sodium pyruvate). The cell suspension was placed in a bottle previously treated with a solution of 1% gelatin. The cells were incubated at 37°C in a water-saturated atmosphere of 5% CO_2_. The culture medium was exchanged on the next day, and then every three days, supplementing with 10 ng/µl epidermal growth factor (EGF) and 20 ng/µl basic fibroblast growth factor (bFGF). Immunohistochemical analysis using anti-CD31 FITC-conjugated antibodies was performed for characterization of endothelial cells.

### Chemiluminescence Assay for Measurement of Nitric Oxide Products (NOx)

For the quantification of NO, 1×10^6^ HUVECs were plated in 6 wells plates with culture medium supplemented with fetal calf serum. After the period of cell adhesion, the culture medium was replaced by medium without serum, followed by 24 h incubation with 1 µM *Bj*-PRO-10c. Then the culture medium was collected and centrifuged for 5 min at 21,000 g to eliminate possible cellular debris, and the supernatant was collected for analysis of NOx. The cells were washed with PBS, lysed for 30 min in ice-cold RIPA buffer (50 mM Tris-HCl, pH 7.5, containing 150 mM NaCl, 1% Nonidet P-40 (NP- 40), 0.5%, sodium deoxycholate, 0.1% SDS, 1 mM DTPA and 10 mM N-ethylmaleimide) and centrifuged for 15 min at 21,000 g and 4°C. NOx determination was carried out as described elsewhere [37]. Intracellular and extracellular media were directly injected into a vessel containing a saturated solution of vanadium (III) chloride in 1N HCl maintained at 90°C. Under these conditions, all nitric oxide-derived products (nitrate, nitrite, nitrosothiol, nitrosamines, and iron-nitrosyl complexes) were reduced and compared with those of standard solutions of nitrate under the same experimental conditions.

### Determination of L-arginine concentration in endothelial cells

L-arginine levels in HUVEC and HUVEC-PE were determined as described elsewhere [20]. Briefly, 1×10^6^ cells in serum-free medium were incubated for 24 h at 37°C with 0.3 µM of *Bj*-PRO-10c. Both, extra and intracellular L-arginine concentrations were determined by amino acid detection analysis using a C18 analytical HPLC column (250 mm, 4.6 mm, 5 µm; Merck).

### Superoxide production

Endothelial cell cultures were transferred into 96 black-well plates with clear bottom (Costar, London, UK) with a density of 5×10^4^ cells per well in serum-supplemented medium to cell adhesion to the plastic surfaces. Then the culture medium was replaced by serum-free medium followed by exposure of cells for 24 h to 1 µM *Bj*-PRO-10c. Superoxide production was detected by oxidation of dihydroethidium to a specific fluorescent product, oxi-ethidium using a Dihydroethidium kit (Invitrogen, CA, USA) [38]. Following a 30-min treatment with the superoxide sensitive probe, the cells were washed twice with buffer (10 mM HEPES, pH 7.4, 144 mM NaCl, 2 mM CaCl_2_, 1 mM MgCl_2_, 5 mM KCl, 10 mM D-glucose). Reading of fluorescence was performed using the automated plate reader FlexStation 3 (Molecular Devices, CA, USA) using excitation and emission wavelengths of 530 nm and 620 nm, respectively.

### Calcium measurements by microfluorimetry

For determination of changes in cytosolic calcium concentration ([Ca^2+^]*_i_*) by microfluorimetry using Flexstation 3, HUVECs were transferred into 96 black-well plates with clear bottom (Costar, London, UK) at a density of 5×10^4^ cells per well in serum-free medium. The cells were then loaded for 60 min at 37°C with the Flexstation Calcium Kit in the presence of 2.5 mM probenecid, as described [18], [39]. The fluorescence was monitored before and after the addition of increasing concentrations of *Bj*-PRO-10c. Samples were read for 120 seconds at 1.52 s intervals with a total of 79 read-outs per well. Fluorescence of samples was excited at 485 nm, and fluorescence emission was detected at 525 nm. Responses were measured by the peak fluorescence intensity following agonist application compared to baseline fluorescence intensities.

### Determination of eNOS and ASS levels by Western-blot

HUVECs were cultured in serum-containing medium to reach 80% of confluence. Then, cells were exposed for 24 h to 1 µM *Bj*-PRO-10c in serum-free medium. After this incubation period, cells were lysed for 20 min in ice-cold RIPA buffer and centrifuged for 5 min at 21,000 g. Fifty µg of each protein sample was separated by a 10% SDS-PAGE. Proteins were transferred from the gel to a nitrocellulose membrane in 0.38 M Tris-HCl, 0.18 M glycine and 20% methanol under constant voltage of 30 V for 12 hours. The nitrocellulose membrane was incubated in TBS-Tween (20 mM Tris-HCl pH 7.4, 0.15 M NaCl and 0.05% Tween) and 5% BSA, followed by three washing steps with TBS-Tween. The membrane was incubated with a 1∶500 dilution of anti-ASS mouse (BD Transduction Laboratories, NJ, USA), a 1∶5,000 dilution of anti-eNOS rabbit (Stressgen Biotechnologies, CA, USA) or a 1∶7,500 of dilution of anti-β-actin mouse primary antibodies (Santa Cruz Biotechnology Inc, CA, USA). Following addition of alkaline phosphatase-conjugated secondary antibodies (Promega Corp., WI, USA), immunostaining was revealed with AP solution (5 M NaCl, 1 M Tris-HCl pH 9.5, 1 M MgCl_2_) containing BCIP (5-bromo-4-chloro-3-indolylphosphate) and NBT (nitro blue tetrazolium). Density of immunostaining was estimated using the ImageJ program (National Institutes of Health, USA). For relative quantification of ASS expression levels, staining intensities were compared to those of β-actin, whose expression should not vary under the used experimental conditions.

### Statistical Analysis

Statistical analysis was performed using one-way ANOVA followed by *Boferroni's* post-test, employing the GraphPad Prism 5 software. Differences were considered significant when *P<0.05, **P<0.01 and ***P<0.001.
